# Modulation of growth characteristics and endogenous hormone by cutting intensity in *Eleutherococcus giraldii*

**DOI:** 10.1371/journal.pone.0327332

**Published:** 2025-08-08

**Authors:** Xufeng Huang, Rui Gu, Guopeng Chen, Chenhui Wang, Yuan Li, Xiaofeng Liang, Rong Dimg, Shihong Zhong

**Affiliations:** 1 School of Pharmacy, Sichuan College of Traditional Chinese Medicine, Mianyang, China; 2 Northwest Sichuan Laboratory of Traditional Chinese Medicine Resources Research and Development Utilization, Mianyang, China; 3 Mianyang Key Laboratory of Development and Utilization of Chinese Medicine Resources, Mianyang, China; 4 School of Ethnic Medicine, Chengdu University of Traditional Chinese Medicine, Chengdu, China; 5 School of Pharmacy, Southwest University for Nationalities, Chengdu, China; NABI: National Agri-Food Biotechnology Institute, INDIA

## Abstract

*Eleutherococcus giraldii* (*E. giraldii*) is a quintessential medicinal plant in traditional Chinese medicine. This study established control, heavy pruning, and light pruning groups to reveal growth indexes and endogenous phytohormones in cultivated *E. giraldii* using enzyme-linked immunosorbent assay (ELISA). Results indicated that light pruning significantly promoted length of new branch elongation, thereby increasing *E. giraldii* branch bark yield. In contrast, heavy pruning inhibited length of new branch elongation and reduced branch bark production. Both pruning intensities enhanced the number and length of newly sprouted clonal plants, facilitating population expansion of *E. giraldii*. The heavy pruning altered the trends of indoleacetic acid (IAA) and abscisic acid (ABA) in apical leaves, as well as IAA and gibberellic acid 3 (GA_3_) in lateral leaves. The light pruning modified the trends of IAA, ABA, and isopentenyl adenine nucleoside (iPA) in apical leaves, as well as IAA and GA_3_ in lateral leaves. Apical leaf IAA promoted new branch growth in *E. giraldii*, while lateral leaf IAA exhibited the opposite effect. iPA played a significant role in eliminating apical dominance and enhancing stress resistance in *E. giraldii*. GA_3_ inhibited new branch growth while delaying leaf senescence. Apical leaf ABA was closely associated with improved stress resistance, whereas lateral leaf ABA primarily inhibited new branch growth. This study provides valuable insights for establishing sustainable logging strategies for *E. giraldii*, protecting wild resources, and offers reference for research on endogenous hormone responses in shrubs under logging interventions.

## 1. Introduction

There are about 35 species of *Eleutherococcus* plants in Asia, mainly distributed in China, Japan and South Korea, and China is home to 26 species and 18 varieties [1,2]. *Eleutherococcus giraldii* (*E. giraldii*) is a perennial shrub of the genus *Eleutherococcus*, which is widely distributed in Sichuan, Gansu, Shanxi and Ningxia provinces in China, and is a typical clonal plant [3]. The 1–2-year-old dried stem bark of *E. giraldii* is commonly used for medicinal purposes. It is officially records in the Standard of Chinese Herbal Medicine in Sichuan Province (2010 edition) and demonstrates therapeutic effects including dispelling wind-dampness, unblocking meridians, and strengthening bones and muscles [4]. Modern pharmacological studies have revealed that *E. giraldii* exhibits anti-inflammatory, anti-arrhythmic, hepatoprotective, anti-tumor, and antiviral properties [5–8], indicating significant pharmaceutical development potential.

Logging is typically employed in forests, arbor woodlands and shrub lands. Based on operational methods, it can be categorized into clear-cutting, shelterwood cutting, selective cutting, etc. Fundamentally, this practice represents a reconstruction of the growth patterns in harvested vegetation, whereby human intervention induces physiological, biological, and ecological modifications in individual plants or plant communities, consequently altering their life cycles [9,10]. *E. giraldii* stem bark is predominantly sourced from wild populations. Traditional understanding suggests *E. giraldii* exhibits ‘enhanced regeneration following harvesting, ‘yet market analyses demonstrate annually tightening supplies accompanied by declining medicinal quality [11]. Our investigations identified that certain harvesters, prioritizing immediate profits, utilize clear-cutting methods while excessively collecting mature stems and bark for medicinal purposes, resulting in substantial damage to wild *E. giraldii* resources and degradation of medicinal quality. Therefore, establishing scientifically validated harvesting protocols is critically important for both resource conservation and maintenance of therapeutic efficacy.

Plant hormones are trace organic compounds synthesized endogenously in plants, which can be translocated from production sites to active sites and elicit physiological responses at low concentrations (<1μmol/L). Various hormones exhibit synergistic or antagonistic interactions, collectively regulating the entire growth process of plants [12,13]. During winter wheat regeneration following mowing, indole-3-acetic acid (IAA) concentrations decrease while cis-zeatin (cZ) levels increase [14]. At the floral bud differentiation stage in *Glycyrrhiza uralensis* Fisch, flowering plants demonstrate significantly higher IAA and zeatin riboside (ZR) contents compared to non-flowering plants, whereas plants with abscised inflorescences exhibit markedly elevated gibberellic acid (GA) and abscisic acid (ABA) levels relative to flowering and fruiting plants [15].

Currently, the characteristic changes of endogenous hormones in *E. giraldii* under pruning disturbances remain unclear, and their correlations with shoot growth parameters are undetermined, with limited research reports available. We examined variations in growth indices and foliar endogenous hormones of *E. giraldii* under both light pruning and heavy pruning treatments, aiming to: (1) evaluate the effects of cutting intensity on plant growth and endogenous hormonal profiles, (2) elucidate the relationships between growth indexes and hormone levels. This study provides scientific evidence for conservation of wild *E. giraldii* populations and sustainable production of medicinal materials with guaranteed yield and quality, while establishing a reference framework for investigating endogenous phytohormone regulation in shrubs under logging disturbance.

## 2. Materials and methods

### 2.1 Plant material, experimental design and growth conditions

**Fig 1:**
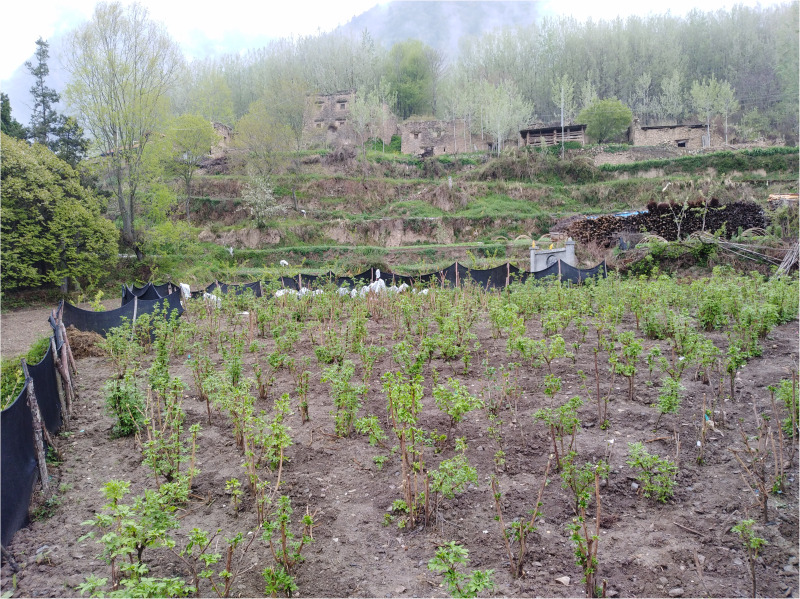
Habitat suitability mapping of *E. giraldii.*

The experimental site is located at Ruowo Village, Sanyilong Township, Mao County (31°47′00.19″ N, 103°30′13.66″ E), Aba Prefecture, Sichuan Province, China, with an elevation of 2600 m above sea level. The site experiences distinct wet and dry seasons, characterized by a mean annual temperature of 11.2°C, ≥ 10°C annual accumulated temperature of 3293.3°C, annual evaporation of 1332.4 mm, and annual precipitation of 494.8 mm (over 80% of which occurs between May and October). The south-facing slope (sunny aspect) features neutral loam soil [16]. The plantation contains more than 200 five-year-old cultivated *E. giraldii* plants, which adequately meet experimental requirements (Fig 1).

**Fig 2:**
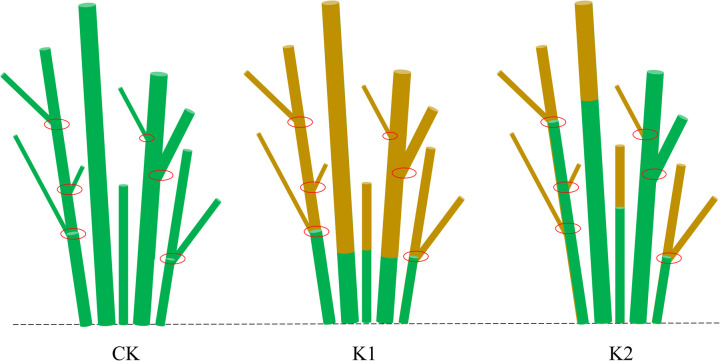
Harvesting regimes for *E. giraldii.* Note: K1 denotes heavy pruning, K2 represents light pruning, red circles indicate stem nodes, brown areas show removed portions, green zones depict retained tissues after pruning, and the dashed line marks ground level.

On May 6th, eighteen cultivated *E. giraldii* shrubs with comparable growth vigor (plant height: 0.8⁓1.2 m; canopy width: 0.4⁓0.5 m) were randomly selected and assigned to three treatment groups (n = 6 per group) using a completely randomized design: (1) heavy pruning group (K1)-complete removal of all stems and branches, (2) light pruning group (K2)-selective retention of 3⁓5 vigorous branches through weak-branch-removal pruning, and (3) control group (CK)-no treatment applied (Fig 2).

### 2.2 Experimental methodology

#### 2.2.1 Quantification of physiological parameters.

Measurements were conducted on five occasions (May 17 [5/17], June 5 [6/05], June 26 [6/26], July 15 [7/15], and July 31 [7/31]) following the methodology of Gu R, et al. [17]. For each experimental shrub cluster, the following parameters were recorded: plant height (PH), number of new branches (NNB), length of new branches (LNB), length of newly emerged clonal ramets per cluster (LCP), and number of newly emerged clonal ramets per cluster (NCP). Herein, NNB refers to the count of newly grown branches that were clearly distinguishable from old branches within the shrub cluster, LNB represents the length of these distinguishable new branches, LCP indicates the length of belowground clonal offspring within the canopy coverage area of the shrub cluster, and NCP denotes the number of these belowground clonal offspring within the same canopy coverage area.

#### 2.2.2 Analysis of endogenous phytohormones.

Following growth parameter measurements, approximately 2 g each of apical and lateral leaves were collected from *E. giraldii* across different groups. Samples were precisely weighed using an analytical balance (±0.01 g accuracy), labeled, and immediately flash-frozen in liquid nitrogen (−80°C) for transport. In the laboratory, samples from the same treatment group were homogenized, packaged in polyethylene bags, and stored at −80°C until analysis. For analysis, 0.3⁓0.5 g aliquots were ground in pre-cooled mortars under low-light conditions with PBS buffer (1:9, w/v) for 3⁓5 minutes. The mortar was rinsed 3⁓5 times with additional buffer, and the combined homogenate was transferred to 10 mL centrifuge tubes. After vortex mixing, samples were extracted at 4°C for 3 hours, followed by centrifugation at 3,500 rpm for 10 minutes (4°C). The supernatant was purified using C_18_ solid-phase extraction columns. Concentrations of IAA, ABA, GA_3_, and iPA were determined using commercial ELISA kits (MEIMIAN, Jiangsu, China) according to the manufacturer’s protocol.

### 2.3 Data processing and statistical analysis

Growth parameters and endogenous phytohormone data were preliminarily processed using Microsoft Office Home and Student 2021, followed by comprehensive statistical analysis with IBM SPSS Statistics 27. Normality (Shapiro-Wilk test) and homogeneity of variance (Levene’s test) were assessed, with non-normally distributed data analyzed using the Kruskal-Wallis test. Results are expressed as mean ± standard deviation (x̄ ± s) for parametric data or median (interquartile range) for non-parametric distributions. Correlation analyses were performed and visualized using Origin 2022, with statistical significance denoted by *P < 0.05 and **P < 0.01.

## 3. Results

### 3.1 Effects of pruning treatments on growth parameters in *E. giraldii*

The results demonstrated that light pruning could effectively promote the elongation of new branches in *E. giraldii*, thereby being more conducive to increasing the branch bark yield of *E. giraldii*. In contrast, heavy pruning significantly inhibited new branch growth, thereby decreasing bark yield. Both light and heavy pruning increased the number and length of newly sprouted asexual shoots, promoting *E. giraldii* population expansion.

**Fig 3:**
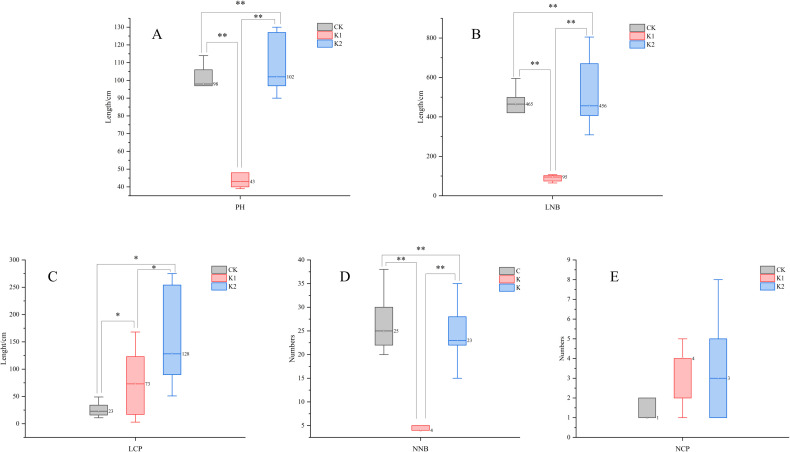
Comparison of growth parameters in *E. giraldii* on 7/31. A)PH; B)LNB; C)LCP; D)NNB; E)NCP. Note: PH, plant height; LNB, length of new branches; LCP, length of newly emerged clonal ramets per cluster; NNB, number of new branches; NCP, number of newly emerged clonal ramets per cluster. *P < 0.05, **P < 0.01.

As shown in Fig 3, compared to the control group, heavy pruning significantly reduced plant height and new shoot length in *E. giraldii* (*P* < 0.01), whereas light pruning significantly increased these parameters (*P* < 0.01). The light pruning treatment resulted in significantly greater plant height and new shoot length than heavy pruning (*P* < 0.01). Both pruning intensities significantly increased the length of newly sprouted clonal ramets compared to the control (*P* < 0.05), with light pruning showing significantly greater values than heavy pruning (*P* < 0.05). Contrary to this, both treatments significantly reduced the number of new shoots relative to the control (*P* < 0.01), though light pruning maintained significantly higher shoot numbers than heavy pruning (*P* < 0.01). Regarding clonal ramet numbers, both treatments showed increases compared to the control, with light pruning demonstrating numerically higher values than heavy pruning, though these differences were not statistically significant.

**Fig 4:**
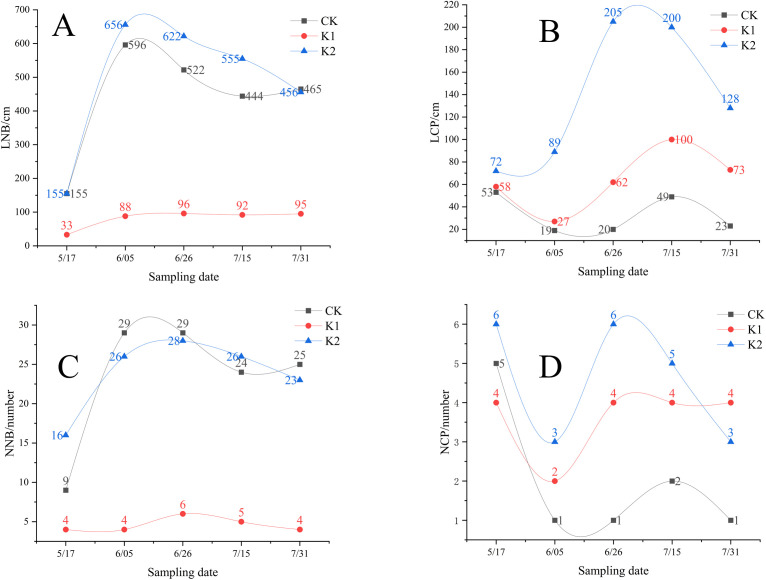
Development trajectories of growth performance metrics in *E. giraldii.* A)LNB; B)LCP; C)NNB; D)NCP. Note: LNB, length of new branches; LCP, length of newly emerged clonal ramets per cluster; NNB, number of new branches; NCP, number of newly emerged clonal ramets per cluster.

Fig 4 demonstrates distinct temporal patterns in growth parameters among treatments: From May 17 to June 5, new branch length increased by 284.5% (control), 166.7% (heavy pruning), and 323.2% (light pruning) year-over-year, with light pruning showing superior elongation throughout May 17⁓July 15 (light pruning > control > heavy pruning). Clonal ramet development revealed treatment-specific peaks: light pruning reached maximum length earlier (June 26, 184.7% increase) compared to heavy pruning (July 15, 72.4% increase), maintaining an overall advantage in ramet length (light pruning > heavy pruning > control). While heavy pruning consistently produced fewer new branches than other groups (May 17⁓July 31), both pruning treatments enhanced clonal propagation, particularly during June 5⁓July 15 when ramet numbers followed light pruning > heavy pruning > control, with both pruned groups exceeding control values.

### 3.2 Temporal fluctuations of endogenous phytohormones in apical leaves

**Fig 5:**
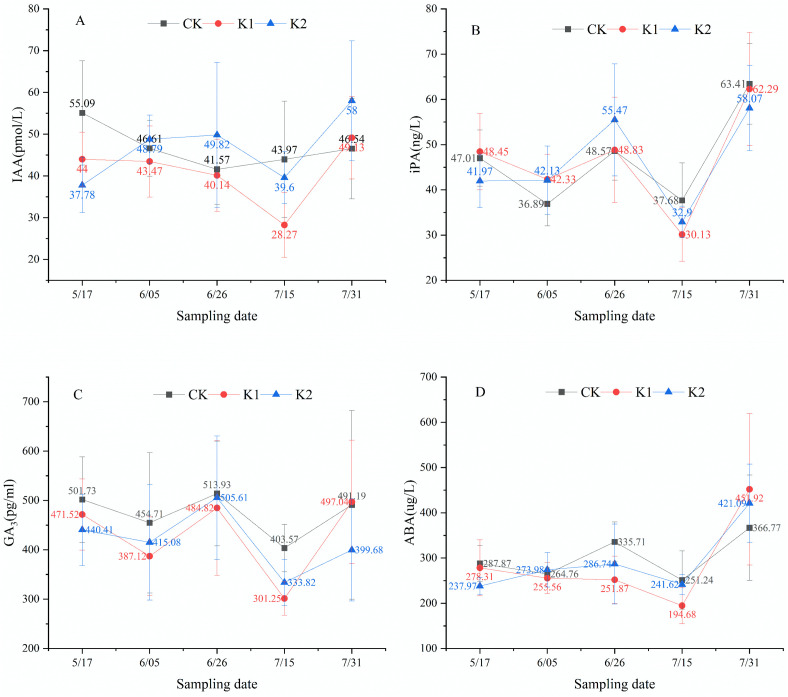
Endogenous phytohormone dynamics in apical leaves of *E. giraldii.* A)IAA; B)iPA; C)GA_3_; D)ABA.

Results indicate that both heavy and light pruning altered the temporal patterns of IAA and ABA in apical leaves, with light pruning additionally modifying iPA dynamics. As shown in Fig 5A, control group IAA levels exhibited a decrease-increase pattern, whereas both pruning treatments showed an increase-decrease-increase trajectory. Fig 5B demonstrates that control and heavy pruning groups displayed iPA fluctuations following a decrease-increase-decrease-increase sequence, contrasting with the increase-decrease-increase pattern under light pruning. Notably, GA_3_ trends (Fig 5C) consistently displayed decrease-increase-decrease-increase cycles across all treatments. For ABA (Fig 5D), controls showed decrease-increase-decrease-increase oscillations, heavy pruning resulted in simpler decrease-increase transitions, while light pruning generated increase-decrease-increase patterns. Statistical analysis revealed no significant differences (*P* > 0.05) in phytohormone concentrations between treatments at any sampling point.

### 3.3 Temporal fluctuations of endogenous phytohormones in lateral leaves

**Fig 6:**
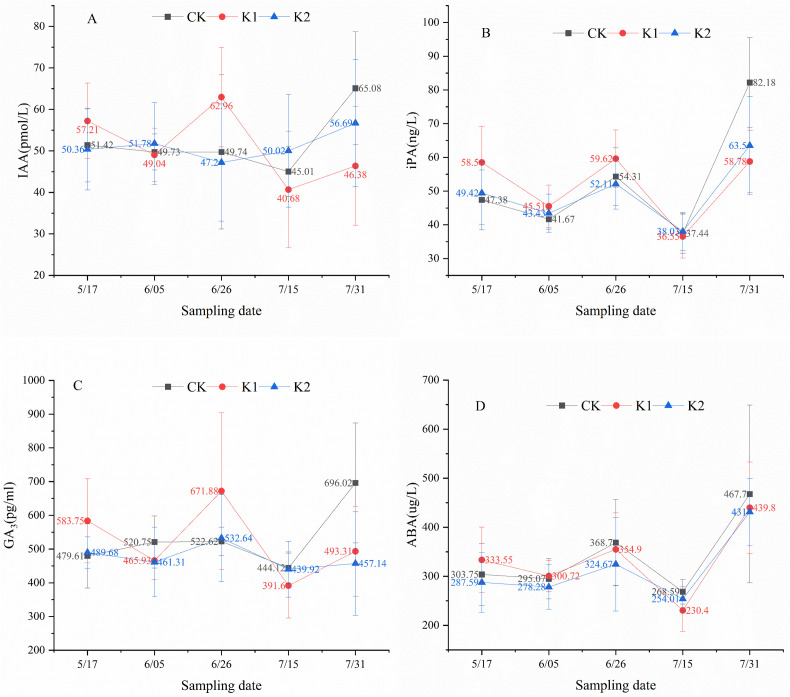
Endogenous phytohormone dynamics in lateral leaves of *E. giraldii.* A)IAA; B)iPA; C)GA_3_; D)ABA.

Results demonstrate that both heavy and light pruning significantly altered the temporal patterns of IAA and GA_3_ in lateral leaves. As illustrated in Fig 6A, the control group exhibited a decrease-increase pattern in IAA levels, while heavy pruning induced a decrease-increase-decrease-increase oscillation, and light pruning resulted in an increase-decrease-increase trend. Fig 6B reveals that all treatment groups (control, heavy and light pruning) showed consistent decrease-increase-decrease-increase fluctuations in iPA concentrations. For GA_3_ dynamics (Fig 6C), controls displayed an increase-decrease-increase pattern, contrasting with the decrease-increase-decrease-increase cycles observed in both pruning treatments. Regarding ABA profiles (Fig 6D), all groups maintained synchronized decrease-increase-decrease-increase oscillations throughout the experimental period. Statistical analysis confirmed no significant differences (*P* > 0.05) in phytohormone levels among treatments at any developmental stage.

### 3.4 Correlation analysis

**Fig 7:**
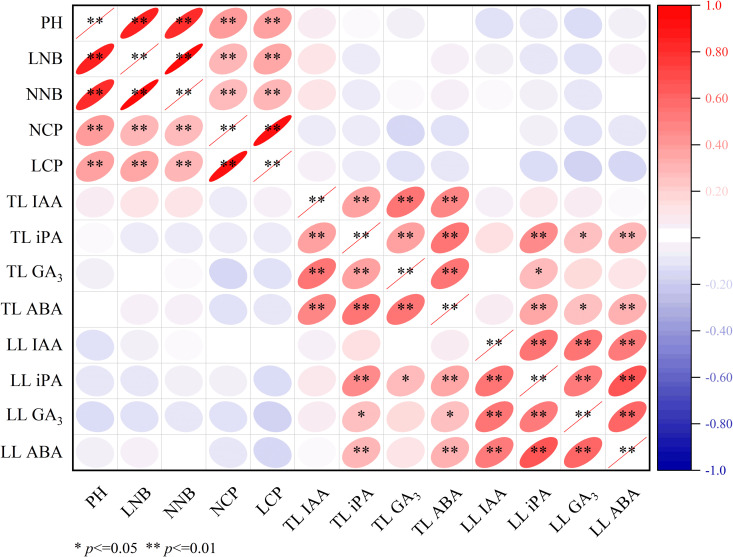
Correlation matrix between growth parameters and leaf phytohormones in *E. giraldii.* Note: PH, plant height; LNB, length of new branches; LCP, length of newly emerged clonal ramets per cluster; NNB, number of new branches; NCP, number of newly emerged clonal ramets per cluster; TL, terminal leaves; LL, lateral leaves (n = 90). *P < 0.05, **P < 0.01.

Fig 7 demonstrates significant (*P* < 0.01) positive correlations between PH (plant height) and key growth metrics including LNB (length of new branches), NNB (number of new branches), NCP (number of newly emerged clonal ramets per cluster), and LCP (length of newly emerged clonal ramets per cluster), with additional positive associations observed between PH and IAA content in terminal leaves (TL-IAA). Notably, LNB showed strong positive correlations with NNB, NCP, and LCP (all *P* < 0.01), while exhibiting positive relationships with both TL-IAA and TL-ABA. Similarly, NNB displayed significant positive correlations with NCP and LCP (*P* < 0.01), along with positive associations with TL-IAA and TL-ABA. The strongest interrelationship was observed between NCP and LCP (*P* < 0.01). In contrast, LCP demonstrated consistent negative correlations with all measured phytohormonal indices.

## 4. Discussion

### 4.1 Effects of logging intensity on growth and medicinal yield in *E. giraldii*

Plant-environment interactions are fundamentally interdependent, with anthropogenic disturbances significantly influencing plant growth and development, inevitably leading to phenotypic modifications. In *E. giraldii*, logging represents the most prevalent harvesting method for medicinal bark production. During June⁓July, stems are cut and debarked, followed by sun-drying. Consequently, bark yield directly determines medicinal output, where greater stem phloem biomass correlates with higher pharmacological productivity [18,19].

Our results demonstrated that the light pruning treatment group exhibited both the highest growth rate and maximum increment in LNB during May 17⁓June 5. However, a paradoxical decline in LNB was observed in the light pruning group from June 5 to July 31, accompanied by reduced NNB in both light and heavy pruning groups during June 26⁓July 31. This phenomenon primarily stems from our operational definition where new shoots were strictly identified as morphologically distinguishable from mature branches. After 50 days of growth, some originally classified new shoots had developed characteristics indistinguishable from mature branches by the June 26 measurement, leading to their exclusion from subsequent records. Notably, the heavy pruning group also showed accelerated new shoot elongation during May 17⁓June 5 compared to other periods. These findings collectively indicate that May 17⁓June 26 represents the critical growth window for *E. giraldii.*

A positive correlation was observed between new shoot characteristics (length and number) and the yield of *E. giraldii* bark, while the clonal propagation capacity was positively associated with both the quantity and length of asexually generated shoots. The heavy pruning group exhibited significantly lower values in both LNB and NNB compared to the control and light pruning groups, indicating that heavy pruning causes substantial damage to vegetative organs and impairs normal growth. Our findings demonstrate that while both pruning intensities can effectively promote population expansion when considering solely reproductive potential, light pruning emerges as the optimal harvesting strategy when medicinal yield is taken into account. These results are consistent with previous findings reported by Ma J, et al. [20].

### 4.2 Regulatory effects of logging intensity on endogenous phytohormones in *E. giraldii*

In clonal plant species, phytohormones function as central regulators within intricate signaling networks, whereby environmental stimuli induce dynamic hormonal interactions that precisely coordinate apical and lateral meristem activities, consequently modifying clonal growth patterns [21].

Following synthesis in shoot apices and young leaves, IAA undergoes basipetal transport via efflux carriers localized at the basal membrane of xylem parenchyma cells, establishing concentration-dependent growth regulation gradients (low concentrations promote growth whereas high concentrations inhibit) [22–24]. Our findings demonstrate that both heavy and light pruning significantly altered IAA distribution patterns in terminal (TL) and lateral leaves (LL). Key temporal patterns were observed from May 17 to June 26: (1) light pruning induced sustained IAA accumulation in TL, contrasting with progressive decline in controls; (2) heavy pruning maintained consistently higher LL-IAA levels versus controls at both timepoints.

Physiological analysis revealed that while both TL-IAA and LL-IAA initially stimulated new branch growth, the characteristic basipetal transport polarity and concentration-dependent inhibition of IAA caused LL-IAA accumulation to ultimately suppress lateral shoot development over time. Specifically, during May 17⁓June 5, light pruning increased IAA in both TL and LL, whereas from June 5⁓26, TL-IAA continued accumulating while LL-IAA declined, indicating attainment of a critical concentration threshold beyond which further IAA secretion inhibits lateral growth. Correlation analyses confirmed these dynamics, showing positive relationships between TL-IAA and new branch growth, but negative correlations for LL-IAA.

iPA is a common cytokinin that promotes lateral branch growth, eliminates apical dominance, and enhances plant stress resistance [25,26]. This study demonstrates that light pruning altered the iPA trend in apical leaves. From May 17 to June 5, both the heavy pruning and control groups exhibited a decline in apical leaf iPA levels, whereas the light pruning group showed a slight increase. A comparison of mean iPA values on May 17 and June 5 revealed the following order: heavy pruning group > control > light pruning group. We hypothesize that a concentration threshold exists between the light pruning and control groups; light pruning reduced apical leaf iPA below this threshold, thereby altering its trend. Furthermore, from June 5 to June 26, iPA accumulation became more pronounced in the apical leaves of the light pruning group, while the heavy pruning group exhibited greater iPA increases in lateral leaves, suggesting that iPA plays a crucial role in suppressing apical dominance and enhancing stress resistance in *E. giraldii*. Correlation analysis further supported a negative relationship between iPA levels and new shoot growth in *E. giraldii*.

GA_3_ plays crucial roles in promoting stem elongation, inducing flowering, and delaying leaf senescence [27–29]. Our results demonstrate that both heavy and light pruning treatments altered the GA_3_ dynamics in lateral leaves. From May 17 to June 5, GA_3_ levels in lateral leaves decreased in both pruning-treated groups but increased in the control group. We propose that this pattern resulted from differential photosynthetic capacities among groups, as evidenced by leaf quantity (heavy pruning < light pruning < control) during this period. The reduced photosynthetic activity in pruning-treated groups likely triggered GA_3_ modulation to enhance photosynthetic efficiency and ensure adequate energy production for plant growth. These findings suggest GA_3_’s vital function in delaying leaf senescence in *E. giraldii*. Correlation analysis further revealed a negative relationship between GA_3_ levels and new shoot growth in *E. giraldii*.

ABA functions in growth inhibition, stomatal closure promotion, and stress resistance enhancement [30–32]. This study revealed that both heavy and light pruning treatments altered ABA dynamics in apical leaves. During May 17⁓June 5, apical leaf ABA decreased in the control group but increased in the light pruning group, while from June 5⁓26, the control group showed increased apical leaf ABA whereas the heavy pruning group exhibited decreased levels. We propose this pattern reflects differential damage to vegetative organs across treatments (control < light pruning < heavy pruning), with pruning-treated groups having higher regenerative growth demands than controls. These results indicate that apical leaf ABA is closely associated with enhanced stress resistance in *E. giraldii*. Furthermore, throughout May 17⁓June 26, lateral leaf ABA levels maintained the order control > light pruning, with all groups showing consistent temporal trends, suggesting lateral leaf ABA primarily inhibits new shoot growth in *E. giraldii*. Correlation analyses confirmed positive relationships between apical leaf ABA and both new shoot length/number, while lateral leaf ABA showed negative correlations with shoot growth.

## 5. Conclusions

*E. giraldii*, a traditional Chinese medicinal plant, demonstrates distinct growth responses to different logging intensities. Light pruning significantly promotes new shoot elongation, thereby enhancing bark production. In contrast, heavy pruning inhibits shoot elongation and indirectly reduces bark yield. Both pruning intensities facilitate the increase in both number and length of newly sprouted clonal stems, promoting population expansion.

Phytohormonal analyses revealed that heavy pruning altered the dynamics of IAA in apical and lateral leaves, ABA in apical leaves, and GA_3_ in lateral leaves. Light pruning affected IAA in both leaf types, ABA and iPA in apical leaves, and GA_3_ in lateral leaves. Notably, apical leaf IAA promoted shoot growth while lateral leaf IAA exhibited opposite effects. iPA played crucial roles in eliminating apical dominance and enhancing stress resistance. GA_3_ suppressed shoot growth and delayed leaf senescence. Apical leaf ABA was closely associated with stress resistance, whereas lateral leaf ABA primarily inhibited shoot growth.

This study elucidates the impacts of pruning disturbance on growth parameters and endogenous hormones in *E. giraldii*, establishing a foundation for further mechanistic investigations.

## Supporting information

S1 Data Research data. (XLSX)
